# Where do we stand in AI for endoscopic image analysis? Deciphering gaps and future directions

**DOI:** 10.1038/s41746-022-00733-3

**Published:** 2022-12-20

**Authors:** Sharib Ali

**Affiliations:** School of Computing, University of Leeds, LS2 9JT Leeds, UK

**Keywords:** Endoscopy, Translational research

## Abstract

Recent developments in deep learning have enabled data-driven algorithms that can reach human-level performance and beyond. The development and deployment of medical image analysis methods have several challenges, including data heterogeneity due to population diversity and different device manufacturers. In addition, more input from experts is required for a reliable method development process. While the exponential growth in clinical imaging data has enabled deep learning to flourish, data heterogeneity, multi-modality, and rare or inconspicuous disease cases still need to be explored. Endoscopy being highly operator-dependent with grim clinical outcomes in some disease cases, reliable and accurate automated system guidance can improve patient care. Most designed methods must be more generalisable to the unseen target data, patient population variability, and variable disease appearances. The paper reviews recent works on endoscopic image analysis with artificial intelligence (AI) and emphasises the current unmatched needs in this field. Finally, it outlines the future directions for clinically relevant complex AI solutions to improve patient outcomes.

## Introduction

Endoscopy is a gold standard procedure for many hollow organs. It is used mainly for disease surveillance, inflammation monitoring, early cancer detection, tumour characterisation and resection procedures, minimally invasive treatment interventions and therapeutic response monitoring. Endoscopic image analysis has started to gain more attention in recent years with a surplus number of endoscopic imaging-based methods being published in computer-aided detection (CADe)^1–5^, computer-aided diagnosis (CADx)^6–11^, and computer-assisted surgery (CAS)^12–16^. Unlike other radiology data (e.g., X-ray, CT, MRI), endoscopy imaging and its analysis is a highly specialised and challenging topic. Endoscopic imaging has multi-factorial dependencies, including large operator dependence (e.g., experience and training), scope-related issues (e.g., imagery quality variability) and underlining scene dynamics (e.g., imminent corruption of frames with severe artefacts, large organ motion and surface drifts^17^). Quality standards in gastrointestinal endoscopic interventions are discussed in several notable guideline studies^18,19^. Some recent works have explored deep learning areas to automate metrics to assess endoscopy quality. These are especially critical in quantifying blind spots^20,21^. While SLAM-based 3D reconstruction was used to generate colonic maps^18^, the length and area of the upper gastrointestinal (GI) cancer precursor, Barrett’s oesophagus, were quantified using deep learning-based depth estimation technique^22^. Similarly, the most crucial task for minimally invasive surgical procedures (e.g., laparoscopy) is understanding and interpreting the underlining scene.

**Fig. 1:**
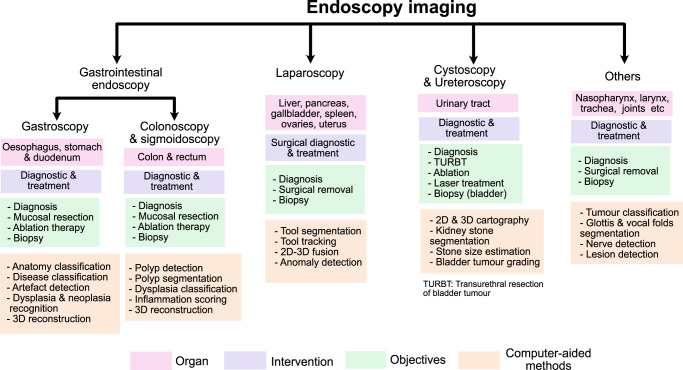
Overview of endoscopic image analysis for surveillance and surgical procedures in the human body. Widely used endoscopic procedures are presented in separate unique categories and subcategories while the rest of the procedures are provided under others. Each is divided into organ of interest, intervention type, objectives and computer-aided methods that are being developed to address some of the objectives presented in these endoscopic procedures.

While a 3D reconstruction of hollow organs is vital, it is difficult to achieve for several reasons, including highly non-linear organ deformation, scene clutter (e.g., fluid running, blood) and occlusion (e.g., fat surrounding liver surgery). Thus, most research is focused on local scene assessment using classification, detection and segmentation methods. Lesion detection and characterisation along with its delineation is a primary focus in GI endoscopy^1–11^. Similarly, targeted stone segmentation and its characterisation is of primary focus in ureteroscopy^23^ and tumour detection^24^ has been explored in cystoscopy. For minimally invasive laparoscopic interventions, surgical tool classification^12^, detection and segmentation^13^, phase recognition^12,14^, segmentation of associated landmarks^15^, and pre-operative 3D volume superimposition on inter-operative 2D laparoscopic^16^ has been an area of focus. A depictive summary of key objectives and various endoscopic image analysis tasks for different endoscopic interventions is presented in Fig. 1.

Most previous review works on artificial intelligence (AI)-driven applications for endoscopic image analysis are published in clinical journals (22 versus only 11 published in the technical journal from 2020 to 2022). The clinical review papers are mostly focused on CADe and CADx systems for lesions in GI endoscopy^25,26^, while the technical review articles are mostly concentrated around the laparoscopic surgery^27,28^. Reviews concerning upper GI (Barrett’s oesophagus, squamous cell carcinoma (SCC) and stomach^25,29^) and lower GI disease (colorectal polyps^26,30,31^, and ulcerative colitis^32,33^) are dominant compared to other organs. In addition, even though some reviews’ titles were generic^26,34^, they only presented studies from GI endoscopic surveillance and did not cover other areas of endoscopic image analysis. To our knowledge, previously published reviews cover only specific endoscopic procedures. They do not engage in a comprehensive summary, including various modalities, varied organ-specific data challenges, nature of lesion and visualisation challenges.

Unlike previous reviews, this paper aims to decipher the path towards clinical integration, which is only possible by putting all endoscopy-related works in one place and pinning down previous and current achievements. In addition, the presented review is concise, highlighting the most important and comprehensive works with similar research collated separately (see Tables 1 and 2). The review illustrates deep learning methods applied to different endoscopic datasets, including cystoscopy, ureteroscopy and nasopharyngeal endoscopy, which were not covered in previous studies. Learning the co-existing challenges and identifying the gaps in each endoscopic procedure is essential to determine the developments required for clinically appropriate and advanced digital healthcare.

**Table 1:** Deep learning for computer-aided gastrointestinal endoscopy: target disease, method, dataset and outcome summaries of selected comprehensive studies.

Type proc.	Organ	Mod.	Target disease	Dataset	Method	Outcome	Similar studies
OGD	O	WL	BE	Train: 494,364 Test: 1704 (669 patients)	Classification^a^^1^—Neoplasia vs NDBE (hybrid ResNet-UNet)	(DS 4) sensitivity: 90%, specificity: 88%, accuracy: 89% (DS 5) sensitivity: 93%, specificity: 83%, accuracy: 88%	Ebigbo et al.^2^ (ResNet100)
OGD	O	NBI	SCC	Train: 6473 images Test: 6671 images and 80 videos	Segmentation^39^ (SegNet)	(Per-image) sensitivity: 98.04%, specificity: 95.03% (Per-frame) sensitivity: 91.5%, specificity: 99.9%	Nakagawa et al.^116^, Sho et al.^117^ (SSD) Everson et al.^5^ (Deep supervision)
OGD	S	WLI	AG	5470 images Train: 70% Test: 30%	Classification^3^ (DenseNet121)	Sensitivity: 94.5%, specificity: 94%, accuracy: 94.2%	Guimarães et al.^4^ (VGG16)
OGD	S	WLI	AG, IM, erosion and hem.	Train: 7326 images Val: 815 images Test: 570 images, 258 external test and 80 videos	Classification^a^^41^ (UNet++, ResNet50)	Accuracy (non AG/AG, atrophy/IM, and erosion/haemorrhage): 88.78%, 87.40% and 93.67% (int. test), 91.23%, 85.81% and 92.70% (ext. test) and 95.00 %, 92.86 %, and 94.74% (video)	Zhao et al.^94^ (UNet)^b,c^
Colon	CR	WL	Polyp	Train: 411 clips Test: 135 clips (videos)	Frame-level polyp/non-polyp classification^42^ (3D CNN, binary)	Sensitivity: 90%, specificity: 63%, accuracy: 76%, FP: 60	Kim et al.^118^ (TL: AlexNet)
Colon	CR	WL, NBI	Polyp	Train: 8641 images Test: 1330 images and 11 videos	Polyp detection with localisation^43^ (YOLO; VGG16 (A1), VGG19 (A2) and ResNet50 (A3))	(A2) sensitivity: 90%, specificity: 95.2%, AUC: 0.991, accuracy: 96%, FP: 7	Yamada et al.^119^ (Faster R-CNN) Klare et al.^c^^95^
Colon	CR	WL, NBI	Polyp	Train: 20,431 images Test: 7077 images (1172 polyps)	Detection^6^ for polyp characterisation (SSD)	(WL) sensitivity: 90%, PPV: 83% (NBI) sensitivity: 97%, PPV: 98%	Lee et al.^120^^d^ Zachariah et al.^121^
Colon	CR	NBI	Polyp	Train: 1100 (adem.) and 1050 (hyp.) Test: 300 images (180: adem. and 120 hyp.)	Classification^9^ for polyp characterisation (AutoML)	Sensitivity: 83.3%, specificity: 91.7%, accuracy: 86.7%	Song et al.^8^ (CNN) Byrne et al.^7^ (CNN)
Colon	CR	WL	IBD (UC)	1651 images Train: 80% Val: 10% Test: 10% and 30 videos	Classification^11^ into MCES scoring (159-layer CNN)	Sensitivity: 83%, specificity: 96% PPV: 86%, NPV: 94%	Ozawa et al.^44^ (GoogLeNet) Becker et al.^a^^45^ (CNN)
Colon	CR	WL	CRC	Train: 464,105 Test: TCH: 20,783, TFCH: 15,441 and TGH: 48,391	Classification^48^ benign/malignant (169-layer DenseNet) (CRCNet)	(Test set: sensitivity, specificity) TCH: 90.4%, 85.3% TFCH: 78.9%, 95.0% TGH: 74.6%, 99.2%	Ito et al.^122^ (AlexNet)

*OGD* oesophago-gastro-duodenoscopy, *DNN* deep neural network, *CNN* convolutional neural network, *WLI* white light imaging, *NBI* narrow band imaging, *PPV* positive predictive value, *NPV* negative predictive value, *O* oesophagus, *CR* colorectal, *IBD* inflammatory bowel disease, *UC* ulcerative colitis, *MCES* Mayo Clinic Endoscopic Subscore, *SSD* Single Shot MultiBox Detector, *A1–A3* architectures from 1 to 3, *TCH* Tianjin Cancer Hospital, *TFCH* Tianjin First Central Hospital, *TGH* Tianjin General Hospital.

^a^Multisite study.

^b^Comparative: DL vs endoscopists.

^c^Prospective study.

^d^Public dataset.

**Table 2:** Deep learning for computer-aided cystoscopy and ureteroscopy datasets: target disease, method, dataset and outcome summaries of selected comprehensive studies.

Type proc.	Organ	Mod.	Target disease	Dataset	Method	Outcome	Similar studies
Cyst.	Bladder	WL/BL	Tumour	Train: 95 patients 2335 frames (benign) 417 frames (cancer) Test: 54 patients	Detection^67^—tumour vs normal (CystoNet)	Sensitivity: 90.9%, specificity: 98.6%	Hashemi et al.^123^ (VGG16) Ikeda et al.^66^ (CNN)
Cyst.	Bladder	BL	Tumour	Train: 10 patients, 196 frames Val: 10 frames Test: 10 frames (total: 216)	Classification^68^ Tumour vs normal (T1) Tumour invasiveness (T2) Grade classification (T3) (Ensemble)	(T1) sensitivity: 95.7%, specificity: 87.84% (T2) sensitivity: 88%, specificity: 96.56% (T3) sensitivity: 92.07%, specificity: 96.04%	NA
Uter.	Ureter	WL	Stone	Train: 127 frames (2 per stone) leave-one-out	Classification^69^—Composition (ResNet101)	Sensitivity (mean): 83.34%, Specificity (mean): 96.5%	Lopez et al.^70^ (Inception)
Uter.	Ureter	WL	Stone	Train: 92 frames Val: 32 frames Test: 30 frames (in vivo human)	Segmentation^71^—Stone and laser (MI-HybridResUNet)	Dice coeff.: 83.47% (stone) 86.58% (laser)	Zachary et al.^124^ (UNet)
Other	Nasopharynx	WL	Tumour	Train: 19,576 frames Val: 2690 frames Test: 5270 frames	Classification/segmentation^73^ (FCN^78^)	Accuracy (mean): 88.7% Dice coeff.: 78% (retrospective), 75% (prospective)	Xu et al.^104^ (Siamese) (WL/NBI)

*T1–T3* Task 1 to task 3, *NA* not applicable, *WL* white light, *BL* blue light.

## Method

**Fig. 2:**
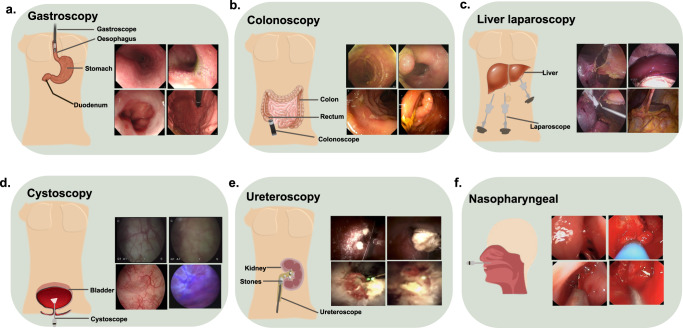
Different endoscopic acquisition systems for various hollow organs. **a** Gastroscopy procedure during which a flexible endoscope is inserted to visualise mucosa in the oesophagus and stomach parts of the duodenum. It can be observed that the scene varies quite a lot depending on the scope location. Similarly, in the top left image, one can observe bubbles surrounding the mucosa. **b** Colonoscopy procedures cover the colon and rectum, during which flexible endoscopes are used to navigate this complex twisted organ. Bowel cleansing is an essential preparation as it can occlude lesions. In most images, the presence of stool is a clear mark of occluded anomaly. **c** During laparoscopy, usually rigid endoscopes are inserted through small incision holes. Images depicting fat surrounding the liver, a clear view of the liver, the presence of tools during surgery and complete occlusion of the liver due to fat are shown. **d** Widely used rigid endoscopes are used for investigating bladder walls that are inserted through the urethra. Conventional white light image modality (first three) and fluorescence image (blue) modality are shown^125^. It can be observed that the top two images are blurry showing little or no vessel structures. **e** Kidney stone removal using ureteroscopy and laser lithotripsy. The difference in texture and surrounding debris (in top) and blood (bottom) for in vivo images^71^. **f** A flexible endoscope enters through the nostrils and can go from the nose up to the throat area and is hence collectively called nasopharyngeal endoscopy. Images (on the left) show a small opening and field of view, along with surgical tools for some cases^126^. The sources of relevant endoscopy images: gastroscopy and colonoscopy images in (**a** and **b** are acquired from Oxford University Hospitals under Ref. 16/YH/0247 and forms part of publicly released endoscopy challenge datasets (EDD2020^127^ under CC-by-NC 4.0 and PolypGen^128^ under CC-by, Dr S. Ali is the creator of both datasets). Liver laparoscopy data are taken from the recently conducted P2ILF challenge^129^ (Dr S. Ali is the creator of this dataset), while cystoscopy and ureteroscopy data are respectively taken from PhD thesis of Dr S. Ali^130^ and a recently published paper of which he is a co-author^71^. Similarly, nasopharyngeal images correspond to publicly available UW-Sinus-Surgery-C/L dataset^126^ with an unknown licence.

Endoscopic procedures are operator-dependent, making them prone to human errors that can result in low adenoma detection rates (ADRs) (e.g., reported in colonoscopy^35^). Other limitations include tumour size, lesion location and technical challenges such as occlusion during minimally invasive laparoscopic surgery^36^. Endoscopic procedures and imaging challenges vary from organ to organ. Multiple rigid endoscopes are used to tackle the limited field-of-view problem in laparoscopic minimally invasive surgery^37^. However, the procedure is very challenging due to other overlapping organs. Similarly, colorectal screening procedures are done using flexible scopes due to colonic peristalsis and bowel movements. There is more evidence of very similar imaging limitations in all these procedures. Bubbles and sometimes food residues are flushed during gastroscopy to clear the mucosa. Also, bowel cleansing is required before imaging the colon. Similarly, the bladder walls are flushed with saline solution during the cystoscopy to make the surface more apparent. Irrigation fluid is used to clear the scene clutter during the kidney stone fragmentation procedure. Scene occlusions are a major challenging factor in nasopharyngeal endoscopy (Fig. 2a–f). In an algorithmic sense, scene clutter affects almost all image analysis algorithms, including today’s AI approaches, i.e., deep learning. It is because it becomes hard to understand the underlying mucosa and difficult to characterise abnormalities that lead to confusing learned networks trained with clean images. For ureteroscopy, floating debris makes kidney stone segmentation and characterisation difficult. Similarly, a decrease in ADR is associated with bowel preparation^38^. Such preparation is also critical and can affect deep learning approaches. The variability in disease appearances from one organ to the other presents comprehensive challenges. However, some of these challenges can be common. For example, imaging quality issues, non-uniform hand motions and organ movements are common in most endoscopic procedures. Similarly, missed lesion detection due to occlusions can be a common limiting factor in all endoscopic procedures. Reviewing these methods in different endoscopic acquisitions aims to understand the most common deep learning approaches and the unique missed opportunities.

**Fig. 3:**
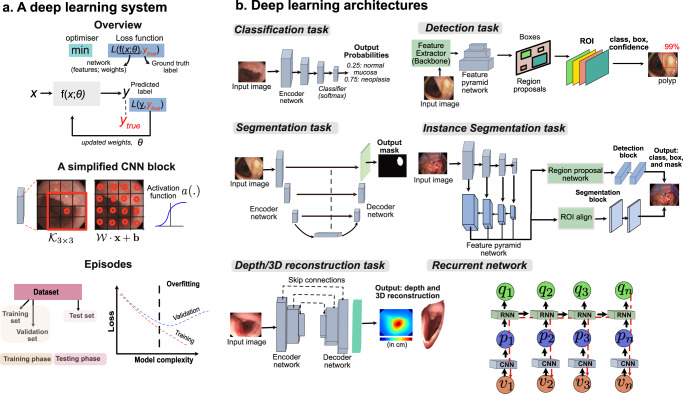
Deep learning system and its widely used designs. **a** A conceptual representation of a deep learning system with an optimiser for minimising a loss function. A simplified convolutional neural network (CNN) block comprising a 3 × 3 kernel and computed weight for each pixel with kernel weights and bias is provided. It also demonstrates a non-linear activation function applied to capture more complex features. The training and test phase consists of split datasets where the validation set is used to guarantee that the learnt parameters generalise and do not overfit the training dataset. A model over-fitting graph is shown that is regulated using a validation set. **b** Some widely used deep learning architectures are shown for various tasks in endoscopic image analysis. For the classification network, only an encoder network is used that is usually followed by a classifier such as *softmax*^3^. For detection, features are extracted using an encoder network, which is then pulled using a region proposal network to predict both the class and the bounding box representations^128^. For semantic segmentation, the encoder features are up-scaled to image size per-pixel classification. Similarly, for the instance-segmentation task, both the region proposals for bounding boxes and per-pixel predictions for masks are used^131^. The idea of a depth estimation network is to understand how far the camera is from an anatomical region providing distances in the real-world coordinate system^22^. Finally, recurrent neural networks (*aka* RNNs) can embed temporal video information to refine current predictions from a CNN network^64^. Here the sequential frame inputs *v*_1_,.., *v*_*n*_ are fed to the CNN network producing visual feature vectors *p*_1_,..., *p*_*n*_, which are then fed to the RNN network. The RNNs output represents the temporal relationship providing context-aware predictions for each frame such that the output for the *n*th frame *q*_*n*_ is dependent on both current and previous frames, i.e., feature vectors *q*(*V*_*n*_) and all other previous feature vectors *q*(*V*_*u*_), *u* < *n*. Both CNN and RNN networks are jointly optimised using boosting strategy. The sources of relevant endoscopy images: gastroscopy and colonoscopy images in (**a** and **b**) are acquired from Oxford University Hospitals under Ref. 16/YH/0247 and forms part of publicly released endoscopy challenge datasets (EDD2020^127^ under CC-by-NC 4.0 and PolypGen^128^ under CC-by, Dr S. Ali is the creator of both datasets). Surgical procedure data are taken from ROBUST-MIS^113^.

Machine learning approaches are data-driven and steered mostly towards minimising (dissimilarity error) or maximising (similarity) a loss function *L* (Fig. 3a). An optimiser, usually a differentiator, is used to find locally optimal values for the computed loss function iteratively. The loss is usually between the predicted labels *y* and the ground truth label *y*_*true*_ (Fig. 3a). Neural networks consist of filters or neurons (aka kernels or weights) that are learnable, unlike classical image processing filters that are predefined. These weights obtained from differently sized kernels (e.g., a 3 × 3 kernel, *K*_3×3_ = {*w*_1_,..., *w*_9_}) are then passed through non-linear activation function *a*(.) that enable them to learn more complex features that otherwise would not be identified. The neural network weights are adjusted based on the optimiser outputs in each iteration. Input samples are mostly processed in batches, for which a complete iteration over all samples is referred to as an epoch during training. The learned model weights are then applied to the test dataset (aka inference or test phase). Most methods optimise loss functions and use validation sets to tune hyper-parameters of the network *θ*. However, such an optimisation can be done for various task settings such as lesion classification, detection and localisation, semantic segmentation (per-pixel classification), instance segmentation (regional box regression and per-pixel classification), depth estimation tasks and others. An overview diagram with known deep learning architectures for neoplasia classification in Barrett’s oesophagus; detection, localisation and segmentation of polyps in colonoscopy; surgical instrument localisation and segmentation during laparoscopic surgery; 3D depth estimation and reconstruction of the oesophagus; and temporal video context inclusion in convolutional neural networks (CNNs) are demonstrated (Fig. 3b).

This review identifies and discusses trends of applying machine learning methods (in particular deep learning) in each organ-specific procedure. Secondly, current gaps leading to future directions are deciphered. The web-based search revealed that most methods optimise the weights using supervised learning tasks consisting of widely used CNNs. These tasks included classification, detection, segmentation, and depth estimation. The literature was selected using search and inclusion criteria provided in the next section. The main focus is to consider recent studies and understand their limiting factors in each imaging procedure and implemented method. We aim to learn methods developed in similar endoscopic techniques and identify ways that can be beneficial in other fields. In future developments, the existing gaps and challenges in endoscopic imaging can allow us to establish a strategic plan and build protocols for reliable and acceptable endoscopic imaging methods.

### Search strategy and inclusion criteria

Medline, Embase, Springer, Web of Science and IEEE Xplore databases were used to search related literature. To focus on organ-specific endoscopic procedural names (e.g., endoscopy, colonoscopy, liver laparoscopy, ureteroscopy) were used. Also, computational studies—machine learning, AI and deep learning—were added together with endoscopic procedure names to condense the search. Most studies after 2018 until early June 2022 are selected for this review. Just for ‘endoscopy deep learning’ with active filters articles, English, 251 papers on Medline and 1740 papers on Embase (as ‘Embase’ did include review papers as well) were found. All duplicates were also removed. Our advanced search using keywords like AI in endoscopy, deep learning for endoscopy, and neoplasia classification revealed 33, 13 and 36 articles, respectively. So, the selected papers are from a ‘basic search’ rather than the advanced search. The basic search revealed a larger number of articles. However, to reduce these, we further applied filters that included borescope, trials, software, photoacoustics, CT, MRI, hardware, simulation, human vs machine studies, micro-ultrasound, whole-slide imaging, radiology etc. Reviews and meta-reviews are also considered from the year 2020 till 2022.

A search on the web of science for laparoscopic surgical interventions included keywords such as laparoscopic liver segmentation and deep learning for laparoscopy. For this, 56 papers, including 36 articles, of which 12 review papers were found. The trend of deep understanding in laparoscopic has grown from 6 papers in 2018 to 21 papers in 2021. Besides specific disease cases, reports that included quality, anatomy classification/recognition, other modalities (e.g., Raman and (hyper)spectral) and depth or 3D reconstruction were also identified. To address the growing trend in clinical and technical communities in gastrointestinal endoscopy, the presented review includes additional method-specific contributions. Eight peer-reviewed conference works have also been added to strengthen the technical contributions in this field.

The presented work has below additional inclusion criteria to make this review more focused, less biased, and reflective of methods towards clinical integration:

- Research papers should have a large patient cohort (compared to previously published works) or at least compared to a few publicly available datasets if it is a technically presented work.
- Research papers should have specific training, validation and test sets reported in the article to reduce bias in studies.
- If the research papers included some novelty but were not comprehensively evaluated on patient data, then such studies were either discarded or were included under method contributions.
- Each rigorously evaluated method was included in the main table. Here, unique modalities, unique target diseases, and individual tasks (e.g., classification, segmentation, detection and localisation) were chosen. At the same time, similar studies are provided in a separate column for interested readers.
- A Section for the AI in other endoscopic procedures that are not widely studied has been included that covers some works on nasopharyngeal, bronchoscopy, and thyroidectomy.
- For the depth map estimation and 3D reconstruction, works are included as a separate section under additional applications as they are not evaluated on more extensive patient datasets. Under the same Section, studies related to quality assurance in endoscopy and anatomical landmark classification are also included to complete this survey.

### Metrics used for the evaluation of methods

#### Computer-aided gastrointestinal endoscopy

Oesophago-gastro-duodenoscopy (OGD) is used to perform upper GI surveillance (including oesophagus, stomach and duodenum). In contrast, colonoscopy and sigmoidoscopy screen lower GI organs, including the colon and rectum. With the recent developments in deep learning, several growths have been in building computer-aided detection and diagnosis systems. Compared to OGD, more research is focused on colonoscopy. Some recent reviews highlighted a few works from selected groups on upper and lower GI^25,26,30^; however, the distinction between train-test dataset or type-of learning-based method used in these studies or both was not presented. A more generic CADe and CADx systems with deep learning (DL) terms were used in the presentation of most found review papers. DL methods for lower GI are presented in^31^; however, these are focused only on colorectal polyps. In this review, training and test data split and the type of algorithm developed for a specific task are clearly and concisely mentioned to give readers an idea of both clinical needs and technical method developments.

For OGD, with the concerning increase of patients with Barrett’s oesophagus, a precursor lesion in the oesophagus has been of prime focus for many current machine learning-based developments. a hybrid ResNet-UNet architecture was used to classify neoplastic and non-dysplastic Barrett’s oesophagus (NDBE)^1^ that provided an accuracy of over 88% on two test datasets. similarly, for SCC in the oesophagus, an encoder-decoder architecture using the VGG16 network for pixel-wise segmentation was used^39^ that reported in sensitivity of 98.04% at 95.03% specificity. Atrophic gastritis (AG) and gastric intestinal metaplasia (IM) are two main stages in the progression of gastric adenocarcinoma^40^, principally caused by helicobacter pylori infection or by autoimmune gastritis. DenseNet121 was trained with 5470 images^3^ to characterise AG, resulting in an accuracy of 94.2% on the test dataset. similarly, UNet++ with ResNet50 was used to classify AG, IM and haemorrhage^41^. For this, 8141 images (4587 patients) were used for training, while 258 external patients and 80 videos were used for the testing stage.

In a colonoscopy, most of these methods target identifying or characterising known cancer precursors, ‘polyps’. Due to their success, most methods implement widely used CNN. For example, 3D CNN for the frame-level binary classification of polyps^42^ with an accuracy of 76%. In contrast, detection methods such as YOLO^43^ and SDD^6^ were used for the localisation and detection of polyps with a much higher accuracy of 96% for YOLO and reported above 90% sensitivity for the SSD approach. AutoML was used by Jin et al.^9^ that allows us to search for efficient neural networks using recurrent and reinforcement learning techniques. The optimised CNN network consisted of normal and reduction cells, which used several operations like separable convolutions and average and max-pooling layers. The searched network obtained an overall diagnostic accuracy for difficult-to-locate polyps was 86.7%. The reported results on performance improvement of novice endoscopists from 73.8% to 85.6% was also reported. Inception^7^ was used to classify polyp and characterise between hyperplastic and adenomatous with a sensitivity of 98% at the specificity of 83%.

There have been, however, attempts to grade inflammation in the bowel, referred to as ‘Inflammatory bowel disease’, focused on both ulcerative colitis (UC)^11,44,45^. Crohn’s disease (CD)^46,47^. IBD remains to have substantial intra- and inter-observer variability in grading disease severity during endoscopy. Several clinically accepted systems for scoring these severities exist that have, to some extent, improved endoscopic score reproducibility and reliability. However, the problem is still vague as these scoring systems include broad definitions. A wide range of deep learning methods has been developed to tackle these issues and minimise operator variability in diagnosis. For UC, Mayo Clinical Endoscopic Scoring (MCES) is the most widely used system for stratifying patients consisting of a 0–3 scoring system from normal (0) to severe (3). An inception V3 model was used to classify between (0 or 1) and (2 or 3)^11^ with 97% accuracy and PPV of 86%. Similarly, a quality control model to distinguish between readable and unreadable frames and a deep learning network based on CNN for UC classification was developed on multicenter datasets reporting an area under the curve of 0.84, 0.85 and 0.85, respectively for MCES ≥1, MCES ≥2, MCES ≥3 (binary classification). CD primarily affects the small bowel, where conventional endoscopes are hard to reach. There are numerous developments in CD scoring using deep learning but for video capsule endoscopy (VCE) imaging. Ulceration and normal mucosa were classified using Xception CNN model training as 5-fold cross-validation showing accuracy over 95% for each fold^46^. A deep learning model that used 169-layered DenseNet^48^ was trained on a large dataset comprising 28,071 images with CRC (3176 patients) and 436,034 non-CRC images (9003 patients). The test was conducted on three unique test sets that included the same and two different centres, demonstrating the trained model’s generalisability with around 75% sensitivity on two unseen test sets.

#### Method contributions in gastrointestinal endoscopy

Development of novel methods on colonoscopy is well documented^31,49^. This can be because of the availability of public datasets for polyp detection and segmentation. These methods are mostly published as conference proceedings and have been included here for completeness. Majority of the current methods for detection and localisation can be divided into multi-stage detectors^50^, single-stage detectors^51^ and anchor-free detectors^52^. In this context, to address the need for real-time polyp detection, Wan et al.^51^ used the YOLOv5 network together with the self-attention mechanism on the top layer of each stage of the feature extraction backbone network to strengthen the informative features showing boost by approximately 2% in Dice score and an improved inference time on two datasets. While most detectors use predefined anchor boxes for localisation tasks, the concept of anchor-free detector^53^ was used to address this, showing a competitive Dice score and improved inference time (nearly 52.6 frames per second) compared to several SOTA methods on four public datasets^52^. Recently, a hybrid 2D-3D CNN network was devised to exploit spatial and temporal correlation of the predictions with marginal gain on video polyp dataset while preserving real-time detection^54^. Detecting abnormality in Barrett’s oesophagus using 3D CNN and convolutional long-short-term memory (ConvLSTM) that enables the capture of spatiotemporal information in videos was also published as a technical contribution^55^.

For segmentation, current developments are based widely on encoder-decoder architectures^56–58^. Tomar et al.^57^ proposed to combine text label embedding as an attention mechanism for effective polyp segmentation and to improve generalisability. During training auxiliary classification task for learning size-related and polyp number-related features was trained and embedded with the segmentation network alongside showing improvement of up to 2% over SOTA methods on four public datasets. Transformer-based networks have also been recently introduced, namely TransFuse^59^ and ColonFormer^60^. TransFuse combined transformers with CNNs in a parallel style allowing capture of both global and low-level spatial details and demonstrated performance gain of nearly 1–2% on five public datasets compared to DL SOTA methods. A recent work showing an improvement over TransFuse was presented as ColonFormer, which used an encoder with mix transformer backbone while the decoder consisted of a pyramid pooling module that allowed to combine layer-wide feature maps of the encoder for a global map. Widely used ad hoc threshold values for final segmentation map prediction were tackled by proposing a ThresholdNet that used confidence-guided manifold mixup as data augmentation enabling optimised threshold learning and showed large improvements (nearly up to 5%) over various SOTA methods.

#### Computer-aided laparoscopic intervention

Surgical intervention review papers and meta-analysis were conducted by 8 out of 33 review papers. Most of these works were published in technical journals. Minimally invasive surgical instrument vision detection, segmentation and tracking algorithms used for the analysis of the images transmitted by surgical robots were presented in ref. ^27^, while DL methods focused on laparoscopic video analysis were conducted in-depth in ref. ^28^. The study^28^ used 32 deep learning approaches. The survey highlighted that nearly half (45%) of the developed methods aimed at instrument recognition and detection, with 20% on phase recognition and nearly 15% on anatomy and action recognition. However, minority papers were on gauze recognition (3%) and surgery time prediction (5%), while the most widely used procedures were cholecystectomy (gallbladder removal surgery, 51%) and gynaecologic surgery (woman’s reproductive system, 26%). In this review, additional papers that have been recently published on anomaly detection, registration, and augmented laparoscopy are added.

An instance segmentation method referred to as ‘mask R-CNN’ was used to segment the uterus, ovaries and surgical tools on the endoscopic images from a gynaecology procedure^61^. ‘SurgAI’ dataset consisted of 461 images. Another study focused on surgical tool detection in laparoscopic videos proposing a multi-label classification named LapTool-Net^62^. LapTool-Net exploited the correlations among different tools and tasks using a recurrent convolutional neural (RNN) network. They used publicly available laparoscopic cholecystectomy datasets, including M2CAI16 and Cholec80. They employed an over-sampling technique for underrepresented classes and an under-sampling of classes with majority samples. An Inception V1 was used for feature extraction with Gated Recurrent Unit (GRU) as RNN blocks, followed by two fully connected classifiers. An autoencoder technique was used as a learnable network to measure the ‘normal’ distribution of the data and detect abnormal events deviating from this distribution as reconstruction error^63^. The training was conducted using the Cholec80 dataset and phantom video data showing recall and precision equal to 78.4%, 91.5%, respectively, on Cholec80 and 95.6%, 88.1% on the phantom dataset. Another similar study on automatic monitoring of tool usage during surgery also exploited temporal context together with visual features (Recurrent network, Fig. 3b)^64^. A recent study used CASENet to predict silhouette and ridge contours of the liver in a 5-patient dataset consisting of 133 images^65^. Even though the paper focused on 3D to 2D contour-based registration, the method was built on the classical computer vision technique using the Perspective-n-Point method with RANSAC for outlier removal.

#### Computer-aided cystoscopy and ureteroscopy

While very few research works directly apply deep learning to endoscopic acquisitions, this field holds enormous potential in developing robust automated methods for lesion detection^66,67^, and characterisation^68^ in cystoscopy. CystoNet^67^ was developed using five fully convolutional networks for pixel-to-pixel prediction and a separate region proposal and ROI pooling layer for bounding box prediction. The training was conducted on 95 patients containing 2335 benign frames and histologically verified 417 frames depicting cancerous tumours. In addition, 54 patient videos with 31 normal mucosa and the remaining 23 patient videos with tumours were used to validate the trained model. Both training and validation data consisted of both white light and blue light (BL) cystoscopy. The study showed that the CystoNet algorithm could identify bladder cancer with per-frame sensitivity of 90.9% and specificity of 98.6%, i.e., the algorithm detected 39 out of 41 bladder cancers. A transfer learning strategy was used for which an ensemble of different pre-trained deep CNN networks (Inception V3, MobileNetV2 network, ResNet50 and VGG16) was fine-tuned and appended with additional layers on top of each network^68^. The study was aimed at classification tasks for BL cystoscopy images, including benign vs malignant tumours, tumour grading (benign, low grade and high grade) and tumour invasiveness (benign, CIS, Ta, T1, and T2). The results demonstrated sensitivity of 95.77% and specificity of 87.84% for malignant lesion identification, while the mean sensitivity and mean specificity of tumour invasiveness were 88% and 96.56%, respectively.

Similarly, for ureteroscopy, kidney stone characterisation^69,70^ and its segmentation for laser lithotripsy (kidney stone fragmentation)^71^ have been developed. For stone characterisation^69^, five different compositions were obtained from a stone laboratory, including calcium oxalate monohydrate (COM), uric acid (UA), magnesium ammonium phosphate hexahydrate (MAPH/struvite), calcium hydrogen phosphate dihydrate (CHPD/brushite), and cystine stones. Sixty-three human kidney stones were used for this study, with at least two images for each stone. Leave-one-out cross-validation method was used to report the results of classification using ResNet101. Specificity and precision for each stone type were (in percentage): UA [97.83, 94.12], COM [97.62, 95], struvite [91.84, 71.43], cysteine [98.31, 75], and brushite [96.43, 75]. Gupta et al.^23,71^ developed motion-based segmentation approach using UNet for both in vivo and in vitro datasets. In addition to the kidney stone, the authors also segmented the laser instrument, stating that it is important to understand the stone’s size and the operating laser distance for laser lithotripsy. The proposed motion-induced HybResUNet improved segmentation results with a reported dice similarity coefficient of 83.47% for stone and 86.58% on in vivo test samples for laser segmentation. The results outperformed baseline networks (e.g., UNet^72^) for both in vivo and in vitro settings.

#### AI in other endoscopic procedures

Some other types of endoscopic images-based deep learning applications include (a) detection of nasopharyngeal malignancies^73^, and segmentation of granulomas and ulcerations on images acquired by laryngoscopy^74^, (b) an end-to-end deep learning algorithm to segment and measure laryngeal nerves during thyroidectomy (a surgical procedure)^75^, and (c) deep-learning-based anatomical interpretation of video bronchoscopy images^76^. A recent review and meta-analysis paper on laryngeal endoscopy^77^ suggested the AI models presented high overall accuracy between 0.806 and 0.997. However, this review did not show details on any AI model and used sample sizes.

Histologically confirmed patient samples consisting of 27,536 images were used for this study, with 19.7% from healthy patients, while the remaining had various pathological diseases, including benign (13.2%) and nasopharyngeal carcinoma (66%). Their overall accuracy was reported to be 88.7% using fully CNNs^78^. Here, a semantic segmentation approach was taken, which yielded in dice similarity coefficient of 0.78 ± 0.24 and 0.75 ± 0.26 on retrospective and prospective test sets, respectively. Similarly, for the laryngoscopy^74^, various lesions were annotated in 127 images from 25 patients to train a UNet architecture showing per-pixel sensitivity of 82% and for granulomas and 62.8% for ulceration. Segmentation of recurrent laryngeal nerve, responsible for human speech, during surgery (thyroidectomy) was achieved using the widely known mask R-CNN (instance segmentation) approach^75^. The dataset included various challenging scenarios such as dim light, close-up, far-away, and bright light and their combinations. The segmentation results ranged from 0.343 to 0.707 at a confidence interval of 95% across 40 subjects. While anesthesiologists commonly use video bronchoscopy during intubation, depth and orientation can be difficult to interpret. Video bronchoscopy decision support system showing the anatomical locations at various rotations was developed using an EfficientNetB1 model with 0.86% classification accuracy (left main branch, right main branch and carina classes), for which 6806 images were used for training while 511 for test^76^.

#### Additional AI-based applications in endoscopy

Apart from focusing on target disease detection and their characterisation, recent literature also shows several method developments related to assisting the quality control of endoscopic screening in GI, mucosal anatomical site detection, and 3D depth estimation or reconstruction for mucosal scene visualisation. Our search showed at least ten papers on endoscopic acquisition quality, four on anatomy classification or detection, and nine on depth map estimation and three-dimensional reconstruction of the mucosa.

Endoscopic quality is a significant bottleneck and can help reduce missed detection rates^18,19^. Works are focusing on both upper GI^21,79^ and lower GI endoscopic procedures^80^ in terms of quality assessment through deep learning. While monitoring blind spots by classifying sites was an indicator of quality control^21^, artefacts such as blur, bubbles, specularity, saturation, and contrast in endoscopic frames were an indicator of the quality in the other study^79^. Off-the-shelf DCNN networks for quality control were used in clinical paper^21^. However, for the methodologically driven framework^79^ the proposition was on combining different weights from the found bounding boxes from a detector YOLOv3 with a spatial pyramid pooling method for a final aggregated quality score and other restoration techniques were proposed for partially defective frames for visual purposes. For scoring the bowel preparation^80^, a deep split-attention residual network was used for training. The test results on 927 images from the external dataset showed an overall accuracy of 96.7%. Similarly, a study focused on understanding the percentage of mucosal visualisation in small bowel during VCE used a simple, fully connected convolution neural network^81^. Similarly, most landmark classification works only applied off-the-shelf CNN networks showing good accuracy in the classification of the landmark sites (e.g., above 90% recall values for 9 out of 11 site classes^82^), widely based on the OGD procedures that include the oesophagus, stomach and duodenum^82,83^.

Depth estimation networks for monocular cases (i.e., a single camera acquisition widely used by most endoscopy systems) were developed^22,84–87^. While a self-supervised learning technique for depth estimation was explored using a Siamese network from a prior SfM tool based on sparse depth estimations from video sequences^84^, recent work by Shao et al.^87^ explored brightness constancy assumption to deal with endoscopic scene illumination variability but again using the self-supervision framework. The former used sinus endoscopic videos demonstrating an absolute relative difference of 0.20 mm while the latter was evaluated on four different public datasets, including a gastrointestinal tract (ex vivo porcine)^86^ where the absolute trajectory error was 0.13 compared to previously published 0.20 on Colon IV^86^. Another work^22^ used a fully supervised depth estimation network to quantify the length of Barrett’s oesophagus for risk stratification. These measurements showed a good correlation with their 3D printed phantom on both length and area, with the relative error below 5% in all cases (maximum relative difference of 0.25 mm on the length and 0.43 mm^2^ on area).

## Current challenges and gaps

### Methodological advancement

In general, most current works in endoscopic image analysis are adopted from prior published computer vision and other medical imaging architectures. Some of these popular networks include Faster-R-CNN^88^, YOLO^89^, UNet^72^, DeepLab^90^ architectures implemented with well-known backbone networks including VGG19^91^, ResNet^92^, and EfficientNet^93^. However, the methods reported in papers, from classification to detection and segmentation, have contributed mainly to their applicability by solving needed clinical problems and extensive evaluation of patient datasets. A technical perspective provided in ref. ^29^ suggested using visual transformers, more hybrid models, the inclusion of explainability in AI models, use of unsupervised and semi-supervised approaches and use of generative models. Reproducibility and test of methods on actual clinical conditions were the major issues raised in another technical review on DL methods for colorectal polyps^31^.

Thus, albeit the reported efficacy of these methods on retrospectively curated data^1,2^, prospective data studies are either not accomplished or have one or a few centre-based analyses^94,95^, making the clinical applicability questionable. The advancement in AI has positively impacted the application opportunities for endoscopic procedural aid and analysis of endoscopic data. On the one hand, many studies published in clinical journals^1,2,39^ have shown their application possibilities. However, they do not compare other architectures rigorously. Novel DL method developments steered towards training on diverse endoscopic datasets, the introduction of explainability of results and more technical works are required to accelerate this field. On the other hand, those published in technical journals do not use comprehensive multi-centre data^12,14,23^. This is because most of these works are primarily focused on using retrospectively collected datasets for algorithmic validation. One can argue that real-world clinical settings can be very diverse compared to the curated datasets. Similarly, data scarcity or lack of annotated data and significant variability in disease cases can lead to data imbalance problems. Some of the recent works published in technical journals have tried to address these important concerns in the field of endoscopic image analysis by including one-shot or few-shot learning approaches^96^, meta-learning approaches^97^, and semi-supervised techniques^98^. However, tackling such problems in prospective clinical cases cannot be pointed out yet. Moreover, some disease cases, such as ulcerative colitis^99,100^ are complex, with highly subtle changes between mild and severe ulcer types, making it harder to classify (accuracy below 85%) using DL-based methods accurately.

### Generalisability of algorithms

Widely used supervised techniques are data voracious and require many human annotations. At the same time, supervised methods can also induce bias due to imperfect labels or different data distribution potentially due to other imaging modalities or even due to different scoping devices used to generate data. An independent and identically distributed i.i.d. dataset is often hard to realise^101^ and does not represent patient variability present in even a selected patient cohort with similar endoscopic procedures and with the same endoscope. Moreover, using these techniques in a stand-alone way with only curated labels from a fixed patient cohort tends to overfit the samples that are predominant in other cohorts or even the same as the variability is likely to change over time. Also, endoscopic imaging includes multi-modal acquisition, varied views, and mucosal changes that can be more varied than any other imaging modality. The free-hand movement of endoscopists to visualise the mucosa or an organ can cause inevitable challenges to the algorithm. In reality, well-curated endoscopic imaging data will not capture these and can affect the algorithm performances in the clinic. Several supervised models have poor generalisability on very close looking but just using a different colonoscopy dataset^102,103^. A recently published work^102^ showed that most DL architectures, including widely used UNet, reported a performance drop of over 20% when a different colonoscopy dataset was used for training and testing. For example, UNet dropped in Dice similarity score from 0.86 when both train and test data were used from the same public dataset to 0.62 when test data differed from the training dataset. As most works perform training, validation and test sets from the same dataset, generalisability studies are very limited in medical image analysis. Thus, this area of research is critical for algorithms to be adaptive to datasets produced in different clinics and varying proportions. Previous studies have shown that the results have been skewed to the centre with more data in training even when combined training is done^103^.

### Exploring multi-modality

Most developed methods use conventional white light imaging. Even though specialised modalities have proven helpful for detecting and diagnosing particular lesions, very little research can be found on more specialised modalities (see Table 1). For example, chromoendoscopy is a well-established medical procedure to enhance the characterisation of GI mucosal tissues^104^. During these procedures, special dyes are used together with optical endoscopy. The observed details can enable the identification of pathology. Similarly, fluorescence cystoscopy^68^ (aka BL cystoscopy or photodynamic diagnosis) in routine clinical practices can improve the detection and visualisation of both papillary bladder tumours and carcinoma in situ lesions compared to standard white light cystoscopy. So, why not exploit these data in addition to the conventional white light modality for more accurate detection and characterisation of lesions? Exploring multi-modal avenues will advance early detection as they contain good visual patterns often not visible in standard procedures (e.g., spectral endoscopic technique^105^). However, advanced techniques also require training and procedural preparation. Thus, learning to adapt from the existing samples and broadly available standard modalities used in daily practices can be a way forward. Domain adaptation and domain generalisation techniques are current unmet needs in this area.

### Validation of algorithms

Algorithmic evaluation is critical for the development of better scientific approaches. These evaluations play a significant role in determining the strength of developed methods for clinical translation. In the context of deep learning techniques, both the test dataset size and the use of evaluation metrics reflecting their performances are essential. It is, however, difficult to establish what number of test samples provide non-skewed results. While unseen test sets determine the generalisability of approaches, most supervised techniques designed are not robust to unseen data distributions^106^. Hence, generalisability assessments or robustness tests are often not included in most papers. Even though standard computer vision metrics are reported in papers (e.g., top 1 accuracy, Sørensen-Dice coefficient, intersection-over-union, precision, and recall), including a metric that assesses the bias between the validation set and test set is required. Such an approach can strengthen the understanding of hyper-parameter tuning and its effect on the unseen test dataset. Also, most current studies neither investigate the data distribution nor illustrate distribution plots demonstrating the variance in data and results. As variance studies are essential to understand the consistency of algorithmic performance, reporting these must be included as a part of algorithmic validation.

### Algorithmic speed and accuracy

With the recent progress in hardware improvement, DL algorithms have been devised that are more accurate and faster simultaneously. However, the need for real-time performance for some tasks, specifically in endoscopic disease detection, diagnosis, and surgical procedures, is more critical. Still, the requirement of high-end hardware to get reasonable speed and accuracy can be economically infeasible at some healthcare centres or challenging to adapt in clinical settings. As a result, network design choices are important to look at either without making performance sacrifices or by appropriately choosing an acceptable trade-off between speed and accuracy is imperative. Faster lightweight networks like PeleeNet^107^ with only 5.4 million parameters with improved accuracy over SOTA MobileNet^108^ and Tiny-YOLOv2^109^ designs can be considered. In addition, model compression methods can enable DL methods to be executed on devices with limited computational capabilities while maintaining the original network’s competitive performance. This method includes pruning, quantisation, knowledge distillation, and network architecture search techniques^110^.

### Methods for subtle lesions

Most methods are built around more obvious cancer or precancerous lesions (e.g., high-grade dysplasia^1,2,68^, polyps^42,43^). The need for identifying subtle early precancerous development thus remains under-explored with conventional endoscopy. In this regard, neoplastic changes at a very early stage, inflammations and other tissue abnormalities that account for the development of serious life-threatening infections should be the focus of novel AI developments. For example, the sensitivity of MCES scoring in patients with IBD is still low with the sensitivity of 83%, even though a binary classification was performed combining 0 and 1 scores as one class and scores 2 and 3 as another class^11^ which is much lower than other lesion classification techniques. However, current developments, even for obvious neoplastic lesions, are definitely of interest as they can reduce subjectivity in treatment procedures and patient management.

### 3D reconstruction, multi-scale and multi-modal registration

While 3D reconstruction of mucosa has been explored for over a decade due to the challenging endoscopic image acquisition, this research direction remains challenging. Deep learning-based depth estimation techniques do have opened an opportunity for mucosal 3D reconstruction^22,84–87^; however, due to the complex endoscopic trajectories and mucosal movements, especially in the hollow organs such as the colon, mucosal visualisation of complete mucosa in 3D remains an open problem. Also, data-driven approaches are yet to be innovated in surgery for pre-operative to post-operative registration.

With several complementary modalities devised and used in routine clinical procedures, including spectral endoscopy, Raman scattering technique, microendoscopy and digital histopathology (optical biopsy), minimal or no effort have been made to explore data-driven methods for multi-scale and multi-modal data fusion techniques. Even though the findings are matched with endoscopy, for example, in spectral endoscopy^105^, these signals are not registered to the region where they are generated.

## Conclusion and directions

In this review, recent deep learning approaches that aimed to minimise inter and intra-observer variability in clinical procedures are highlighted. These developed methods primarily focused on automatic lesion detection, characterisation, localisation, segmentation, surgical support and 3D visualisation or measurement. We also outlined current challenges and gaps in these AI-based approaches and their validation strategies. Research papers in the endoscopic community are primarily steered largely on applying methods from the vision community, demonstrating grim progress in problem-based unique method developments and a lack of comprehensive multi-centre studies. Shallow validation of algorithms and race to publish has mainly affected the quality of research in this area. Also, current needs are ignored due to this, and most apparent lesions are picked repetitively instead of working on subtle flat or sessile lesions or early-neoplastic changes. Taking a bold stand, below future directions are proposed with the assumption that these propositions will help develop unbiased, advanced, and clinically practical AI approaches that are today’s needs.

### Mitigating gaps in AI approaches by learning from challenges dealt with in other endoscopic procedural types

Even though each endoscopic procedure is unique, methodological advances are more progressive and repetitive in one than the other. While this opens up an opportunity for algorithm developers where applications are still uncommon, the lack of dataset and little participation of clinical experts have made these procedural types less attractive. However, there is a clear opportunity and need for similar developments of these computer-assistive technologies in all endoscopic procedures to improve patient care. For example, tackling gastrointestinal pathology using AI has an overwhelming number of papers^25,31^ (see section ‘Computer-aided gastrointestinal endoscopy’). In contrast, despite cystoscopy and ureteroscopy procedures being equally challenging, literature shows minimal work reported so far^66,71^.

### Multi-modal and multi-scale data integration

The questions are ‘What is challenging to assess in routine clinical procedures’; and ‘what AI should pick in terms of detection and diagnosis?’ Are lesions easy to locate by a trained clinical fellow, or is it challenging to find even by an expert (e.g., inconspicuous lesions)? Specialised algorithmic developments and more expert time in curating data are vital for the latter case. Alongside this, complementary modalities can play a significant role in assessing hidden and subtle lesions that can harm patients^20,21^. While human vision is limited, and the mind can only interpret what it can make sense out of the eye, computers can solve more complex data such as multi-modal and multi-scale data signatures^105^. Multi-modality is the key to answering the above questions and is the way forward in tackling hard-to-find lesions. At the same time, multi-scale can provide more detailed characterisation to understand it better, which can complement the strength of AI in this field.

### Multi-centre data validation

Method validation should be first assessed on a multi-centre and heterogeneous retrospective dataset. Since deep learning is very susceptible to data distribution, a model trained on one particular imaging device or a population can lead to market monopoly and limited access to advanced healthcare systems. As a result, it significantly impacts society and the economy. Encouraging the research community to include generalisability assessments is the only way towards a more secure and desirable ecosystem of method development. While access to data due to privacy concerns can make the assessment difficult, the way forward in this direction is to use a federated learning approach that enables assess of multi-centre data and help in the development of generalisable methods that can be both used to build and validate methods^111^.

### Clinical validation

Access to more publicly available clinically acquired datasets consisting of curated and real-world data can be critical for algorithmic development and its adaptation to clinical scenarios. Some examples of these datasets include colonoscopic videos and related annotations in LDPolypVideo^112^ and ROBUST-MIS dataset for surgical tool detection, segmentation and tracking^113^. Similar comprehensive datasets can help assess methods and encourage technical advances towards translational feasibility. Furthermore, to assess the usability in clinical scenarios, the developed approaches can also be encouraged to perform prospective studies at a few community centres. Ideally, clinical studies in local centres should be acceptable to understand translational feasibility and limiting factors.

### Environmental aspect

With the growing deep network architectures and analysis of larger data volumes (e.g., videos in endoscopy), there has been an increasing energy consumption and carbon footprint of DL methods that need to be addressed by the community^114^. The editorial teams should be encouraged to assess each submitted work involving AI-based approaches using additional metrics before sending it for peer review. These metrics can include: (1) papers that use larger DL networks which are impracticable in clinical settings and are responsible for high carbon footprint^115^ should be encouraged to perform model compactness strategies and justify the model selection choices, (2) the importance of conducted work should be weighted by assessing the comparison of method novelty versus state-of-the-art methods, and (3) the robustness versus test run time experiments should be assessed. The submitted works should clearly outline these parameters in their submitted paper abstract and provide a mandatory checklist as an additional file during submission.
